# Third BIR domain of XIAP binds to both Cu(II) and Cu(I) in multiple sites and with diverse affinities characterized at atomic resolution

**DOI:** 10.1038/s41598-019-42875-7

**Published:** 2019-05-15

**Authors:** Shen-Na Chen, Tian Fang, Jing-Yang Kong, Bin-Bin Pan, Xun-Cheng Su

**Affiliations:** State Key Laboratory of Elemento-Organic Chemistry, Department of Chemical Biology, College of Chemistry and Collaborative Innovation Center of Chemical Science and Engineering (Tianjin), Nankai University, Tianjin, 300071 China

**Keywords:** Metalloproteins, Metalloproteins

## Abstract

The X-chromosome linked inhibitor of apoptosis, XIAP, is mainly known as the inhibitor of caspases by direct interaction with caspases with its baculoviral IAP repeat (BIR) domains. XIAP has three BIR domains and each BIR domain contains a zinc binding site, normally known as zinc finger motif. Recent studies showed that XIAP is involved in copper homeostasis in cells and the BIR domains bind copper ion. However, structural details of the second and third BIR domain, BIR2 and BIR3, in XIAP, with copper as well as the binding modes are not known. In the present work we characterize the structural properties of BIR3 in solution by high resolution NMR and other biophysical techniques. The interaction of BIR3 with copper both *in vitro* and in cell lysates was analyzed. Our results show that BIR3 is able to form stable complexes both with Cu(II) and Cu(I), whereas zinc binding site is not affected and zinc retains tightly bound in the zinc finger during these interactions. Surprisingly, BIR3 has multiple binding sites for Cu(II) and Cu(I) but with varied binding affinities. In addition, the solvent exposed Cys351 is readily oxidized by Cu(II) resulting an intermolecular disulfide bond either between two BIR3 molecules or a mixed disulfide bond with glutathione in cell lysates.

## Introduction

Copper is one of the essential trace metal ions in living systems and the homeostasis of copper ion in cells is highly important to sustain the cellular functions of copper binding proteins. Recently, it is reported that the X-chromosome linked inhibitor of apoptosis (XIAP) is involved in the copper ion homeostasis. In general, XIAP is a direct inhibitor of caspases and is considered as a potential drug target for therapy of cancer^1,2^. XIAP with an amino acid sequence of 497 residues has three baculoviral IAP repeats (BIR) domains, an ubiquitin-associated domain (UBA) and a really interesting new gene (RING) domain at the C-terminus. Each BIR domain has a zinc binding motif CCHC. The RING domain contains a CCCHCCCC zinc binding motif that binds two zinc ions. In contrast, UBA has no zinc binding site. XIAP directly binds caspases 3 and 7 specifically and is mainly recognized as the inhibitor of apoptosis. The first BIR domain in XIAP, BIR1, is involved in the NF-κB pathway via interaction with TAB1, and BIR2 domain mediates the inhibition of caspase-3 and 7 and BIR3 interacts with caspase-9^3–7^.

**Figure 1:**
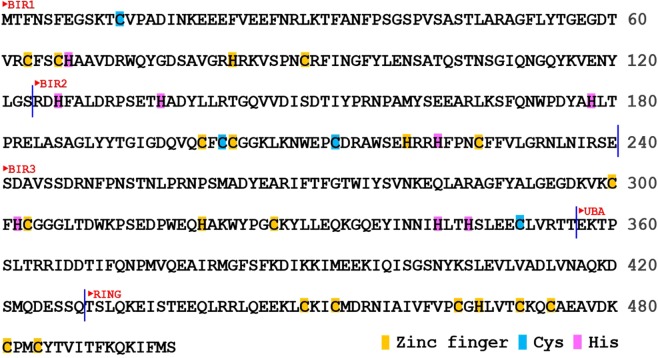
Amino acid sequence of XIAP. The individual domains containing BIR domain, UBA domain and RING domain, are marked and each domain is separated by vertical blue lines. Cys and His in zinc fingers are colored yellow, and other Cys in blue and His in magenta, respectively.

The homeostasis of copper ion in living systems is tightly controlled^8^ and the concentration of copper in human cells is tuned by copper chaperones and the related copper binding proteins^9^, and imbalance of copper concentration results in human diseases, for example, Menkes or Wilson’s diseases^10^. In addition to the generally known copper binding proteins^9^, XIAP is recently discovered that it participates in cellular copper homeostasis via direct interaction with copper ions^11–13^. XIAP has several cysteines and histidines including the zinc binding motif in BIR and RING domains that potentially interact with copper (Fig. 1). Binding to copper changes the conformation of XIAP, leading to instability of the protein^12^. Following studies showed that copper (I) replaces zinc ion from the zinc finger in BIR2 and BIR3, and additional cysteines in the BIR domains are also involved in copper binding^13^. In contrast to BIR2 and BIR3, recent report showed that BIR1 only interacts with copper with the solvent cysteine 12 in the N-terminal domain by high resolution NMR^14^. Copper(II) oxidizes cysteine 12 resulting in disulfide bond formation between two BIR1 domains whereas copper(I) only forms coordination complex with the thiol group of cysteine 12. It is noted that both copper (II) and copper (I) could not replace zinc ion from the zinc finger in BIR1^14^.

BIR3 differs from BIR1 and BIR2 in the zinc binding motif as it contains a histidine 302 that sits among the zinc finger motif (C300, C303, H320 and C327). The presence of additional histidine in the zinc finger in BIR3 raises the possibility whether histidine 302 interacts with copper or affects the competition reaction between copper and zinc with zinc finger motif. In addition, BIR3 in XIAP interacts with COMMD1 in the process of COMMD1-mediated copper levels in cells^11^. COMMD1 was proposed to bind copper(II) with H101, M110 and H134^15^. In addition, BIR3 interacts with the second domain of copper chaperone of SOD (CCS) in the ubiquitination of CCS^16^. These studies indicate that interaction of BIR3 with copper ion is highly important to understand how copper levels is regulated by the protein interactions between XIAP and COMMD1 as well as CCS. However, these interactions of BIR3 with copper ion and the structural details in these interactions are still unknown.

In the present study, we aimed to characterize the interaction of BIR3 with copper(II) and copper(I) using high resolution NMR spectroscopy together with other biophysical methods. Contrary to the previous reports^13,14^, our results show that BIR3 differs greatly from BIR1 in the interaction with copper. We found that BIR3 binds both Cu(II) and Cu(I) in formation of stable complex. Surprisingly, BIR3 endows two interaction sites for Cu(II), of which H343 and H346, and C351 are the main contact residues. In contrast, BIR3 contains three major binding sites for Cu(I). The side chains of H343 and H346 bind both Cu(II) and Cu(I) but form more stable complex with Cu(I). In agreement with the previous findings in BIR1^14^, zinc is tightly bound in zinc finger during this interaction and overall structural fold of BIR3 is essentially unchanged when copper is bound.

## Results and Discussion

### Backbone assignment of BIR3

The construct of BIR3 containing residues 241–356 in XIAP was expressed in *E*. *coli* and purified from inclusion body by denature and refold process. In general, 20 mg was made from 250 mL M9 media. We found that zinc is important to stabilize the overall folded structure of BIR3 and removal of zinc ion from BIR3 with excess of EDTA results in denatured form as evidenced by ^15^N-HSQC spectrum (data not shown).

**Figure 2:**
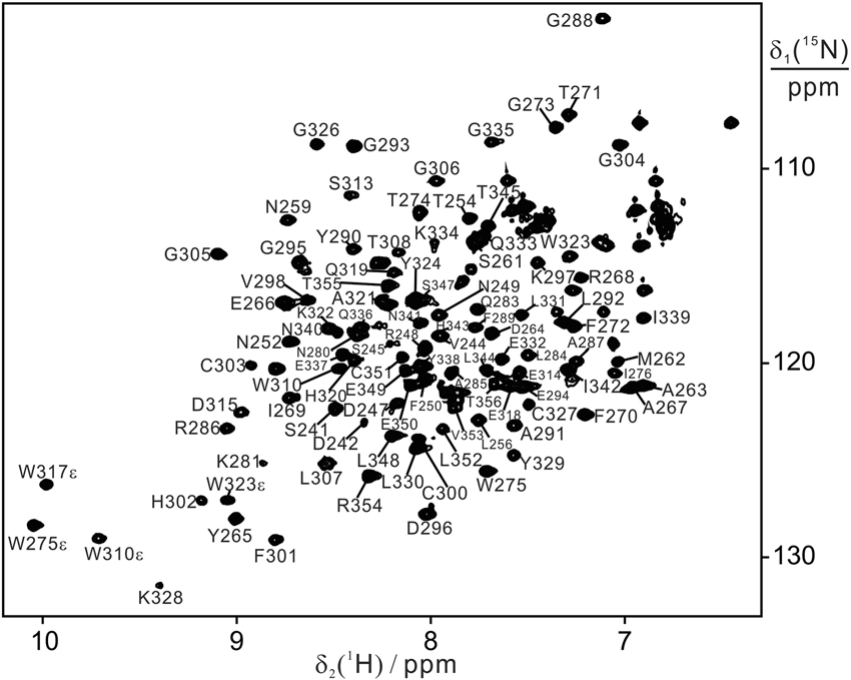
^15^N-HSQC spectra of BIR3 in solution. The NMR spectrum was recorded for 0.1 mM wild type BIR3 (241–356) in 20 mM Bis-Tris buffer at pH 6.5 and 298 K with a proton frequency of 600 MHz. The cross-peaks with assignment were labelled.

Similar to the published data^17^, BIR3 presents a well dispersed ^15^N-HSQC spectrum in solution and the backbone assignment was made from triple resonance experiments of CBCANH and CBCA(CO)NH with the assistance of NOESY-^15^N-HSQC spectrum. All the cross-peaks of backbone amide groups in the ^15^N-HSQC spectrum were assigned (Fig. 2). Compared with published assignment of free BIR3, residues in the loop regions of 276–280, 308–314 were mostly assigned except D309. In addition, the cross-peaks of S253, N255, Y277, E282 and W317 were not observed.

### Interaction of BIR3 with Cu(II)

#### Cu(II) oxidizes BIR3 C351 both *in vitro* and in cell lysates

**Figure 3:**
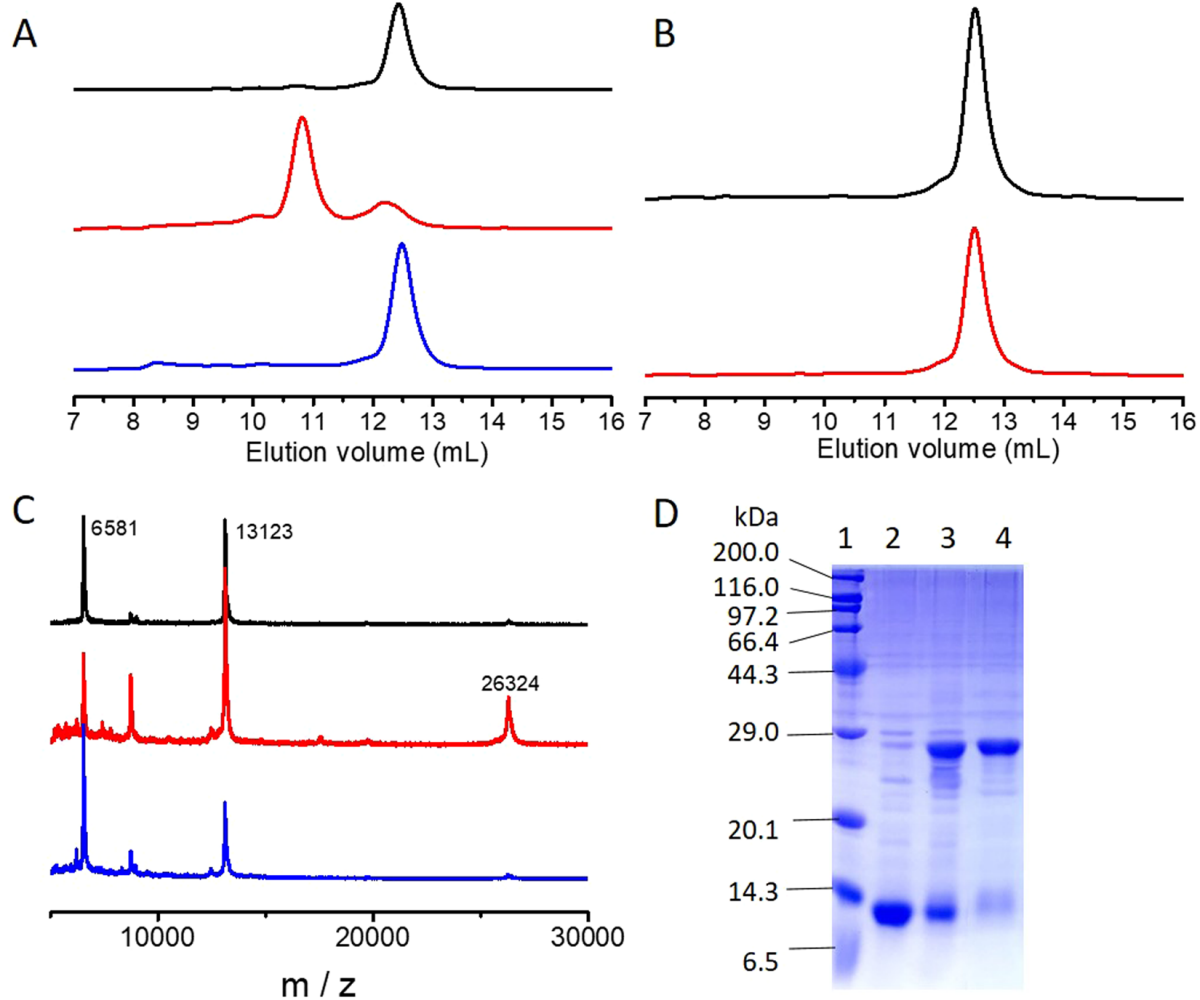
Interaction of BIR3 with Cu(II) analyzed by SEC and MALDI-TOF spectrometry. (**A**) Results of SEC experiments recorded for the mixture of wild type BIR3 before and after addition of Cu(II): 0.1 mM BIR3 (black); mixture of 0.1 mM BIR3 and 0.1 mM CuSO_4_ (red); mixture of 0.1 mM BIR3 and 0.1 mM CuSO_4_ after treatment with 0.6 mM DTT (blue). (**B**) Results of SEC experiments recorded for the mixture of BIR3 C351S mutant before and after addition of Cu(II): 0.1 mM BIR3 C351S (back); mixture of 0.1 mM BIR3 C351S and 0.1 mM CuSO_4_ (red). (**C**) MALDI-TOF mass spectrometry of the SEC fraction recorded for the reaction mixture of BIR3 and CuSO_4_. Top: free BIR3 as reference; middle: fraction with larger molecular weight (first fraction in **A**); bottom: fraction with similar weight of BIR3 (second fraction in **A**). (**D**) SDS-PAGE results run for the different protein samples from left to right lane. Lane 1: molecular marker; 2: free BIR3; 3: BIR3 treated with Cu(II) (also in Fig. S3); 4: fraction with large molecular weight from SEC experiment for the reaction mixture of BIR3 and Cu(II).

In addition to C300, C303 and C327 in the zinc finger motif, BIR3 contains a solvent exposed C351 at the flexible C-terminus (Fig. 1). Addition of copper(II) sulfate into the solution of BIR3 resulted in line-broadening effects for many residues as shown in the ^15^N-HSQC spectrum (Fig. S1). The cross-peak attenuation caused by copper(II) was eliminated by addition of DTT, suggesting the interaction of BIR3 with copper(II) can be reversed by DTT. The MALDI-TOF spectrometry indicated that interaction of BIR3 with Cu(II) generated dimeric BIR3 complex in solution, implying that BIR3 was oxidized by Cu(II) (Figs 3 and S2).

To further characterize the interaction of BIR3 with Cu(II), we performed size exclusion chromatography (SEC) experiments. For the reaction mixture of BIR3 and Cu(II), a protein fraction with larger molecular weight was observed, and it was the dimeric BIR3 as confirmed by MALDI-TOF and SDS-PAGE gel. In contrast, SEC experiment showed that the reaction mixture of BIR3 and Cu(II) after treatment with excess of DTT presented similar elution time as free BIR3. We assumed that C351 might be oxidized by Cu(II) resulting a disulfide bond between two BIR3 complexes on the basis of our previous result of BIR1 and Cu(II)^14^. The solvent exposed Cys12 in the N-terminal flexible segment in BIR1 has very low redox potential and is readily oxidized by Cu(II)^14^. To prove this concept, we made C351S mutant and performed NMR titration and SEC analysis. We did observe cross-peak intensity attenuations of BIR3 upon addition of Cu(II) (to be discussed in the following sections). In contrast, the SEC experiment indicated that the reaction mixture of BIR3 C351S and Cu(II) eluted rather similar to free BIR3 C351S, suggesting that the overall molecular size of BIR3 C351S remains essentially unchanged or BIR3 C351S is still monomer after treatment with Cu(II) (Fig. 3).

To evaluate the interaction of BIR3 with Cu(II) in cells, in-cell NMR spectra were recorded in *E*. *coli*. Unfortunately, we could not observe any dispersed NMR signals in the ^15^N-HSQC spectra recorded from the live *E*. *coli* cells (Fig. S4), suggesting that BIR3 interacts with cellular components that broaden the NMR signals. Since GSH is highly abundant in cells, we proceeded to analyze the interaction of BIR3 with Cu(II) in the presence of GSH. MOLDI-TOF experiment showed that a fraction corresponding to the molecular weight of disulfide bond bridged BIR3-GSH complex was produced after treatment of 0.1 mM BIR3 with 0.1 mM Cu(II) in the presence of 0.8 mM GSH. Notably, the abundance of dimeric BIR3 was barely increased compared with free BIR3 in the mass spectrum. These data suggested that in the presence of GSH, formation of disulfide bond between BIR3-GSH prevailed over two BIR3 molecules upon treatment with Cu(II).

The interaction of Cu(II) with BIR3 was elevated in *E*. *coli* cell lysates by NMR titrations. In contrast to the in-cell NMR spectrum, BIR3 in *E*. *coli* lysates presented well dispersed ^15^N-HSQC spectrum. No significant chemical shift changes of BIR3 between in NMR buffer and in cell lysates were determined (Fig. S4). Addition of Cu(II) into BIR3 in cell lysates generated no significant changes on the NMR signals up to 0.1 mM copper sulfate was loaded. It is noted that higher concentration of Cu(II) (0.3 mM) generated cross-peak intensity attenuations for many NMR signals in the ^15^N-HSQC spectrum (Fig. S4).

**Figure 4:**
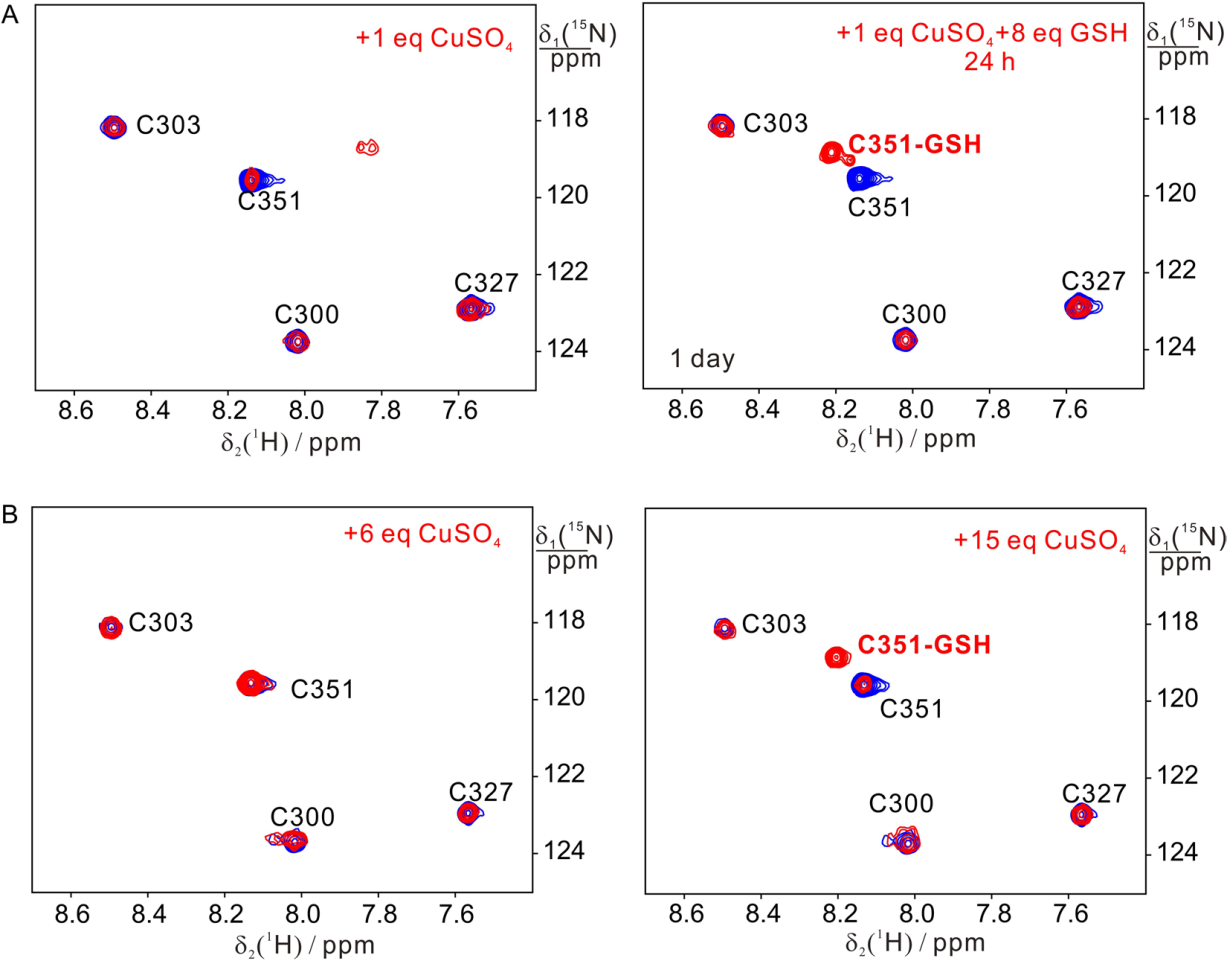
Superimposition of ^15^N-HSQC spectra recorded for ^15^N-labeled Cys BIR3 H302A/H343A/H346A in the absence (blue) and presence of Cu(II) or Cu(II) and GSH (red) *in vitro* and in *E*. *coli* lysates. (**A**) NMR spectra were recorded in 20 mM Bis-Tris buffer, pH 6.5. From left to right: 0.1 mM BIR3 and 0.1 mM CuSO_4_ (red), 0.8 mM GSH was added into the mixture of 0.1 mM BIR3 and 0.1 mM CuSO_4_ and the reaction mixture was incubated for 24 h (red). (**B**) NMR spectra were recorded in *E*. *coli* cell lysates. From left to right: 0.1 mM BIR3 and 0.6 mM CuSO_4_ (red); 0.1 mM BIR3 and 1.5 mM CuSO_4_ (red). It was noted that a new cross-peak of C351 was produced that was due to the disulfide bond formation between BIR3 C351 and GSH.

To differentiate whether C351 is coordinated or oxidized by Cu(II) in cell lysate, we attempted to make selectively ^15^N-Cys labeled BIR3 to simply the NMR spectra and performed the interaction of ^15^N-Cys BIR3 *in vitro* and in cell lysates. In order to unambiguously assign the residues interacting with Cu(II), ^15^N-Cys labeled H302A/H343A/H346A mutant was made and purified, and the interaction with Cu(II) was monitored by ^15^N-HSQC spectra. As shown in Fig. 4, addition of Cu(II) into the solution of BIR3 produced negligible chemical shift perturbations on the cysteine residues (C300, C303 and C327) in the zinc motif whereas the cross-peak intensity of C351 was greatly attenuated. Notably, one small cross-peak with broader linewidth was produced after one equivalent of Cu(II) was added. Treatment of the above reaction mixture with 8 equivalents of GSH resulted in a strong new cross-peak close to C351 and the broader cross-peak generated by addition of Cu(II) disappeared. In addition, the cysteine residues in zinc finger experienced no significant changes. MALDI-TOF mass spectrometry indicated that one new species corresponding to the molecular weight of BIR3-GSH was determined. Similar results were also observed for the reaction mixtures of BIR3 with Cu(II) in *E*. *coli* cell lysates when excess of Cu(II) was added (Figs S4 and 4).

#### H343 and H346 are the major Cu(II) binding sites in BIR3

**Figure 5:**
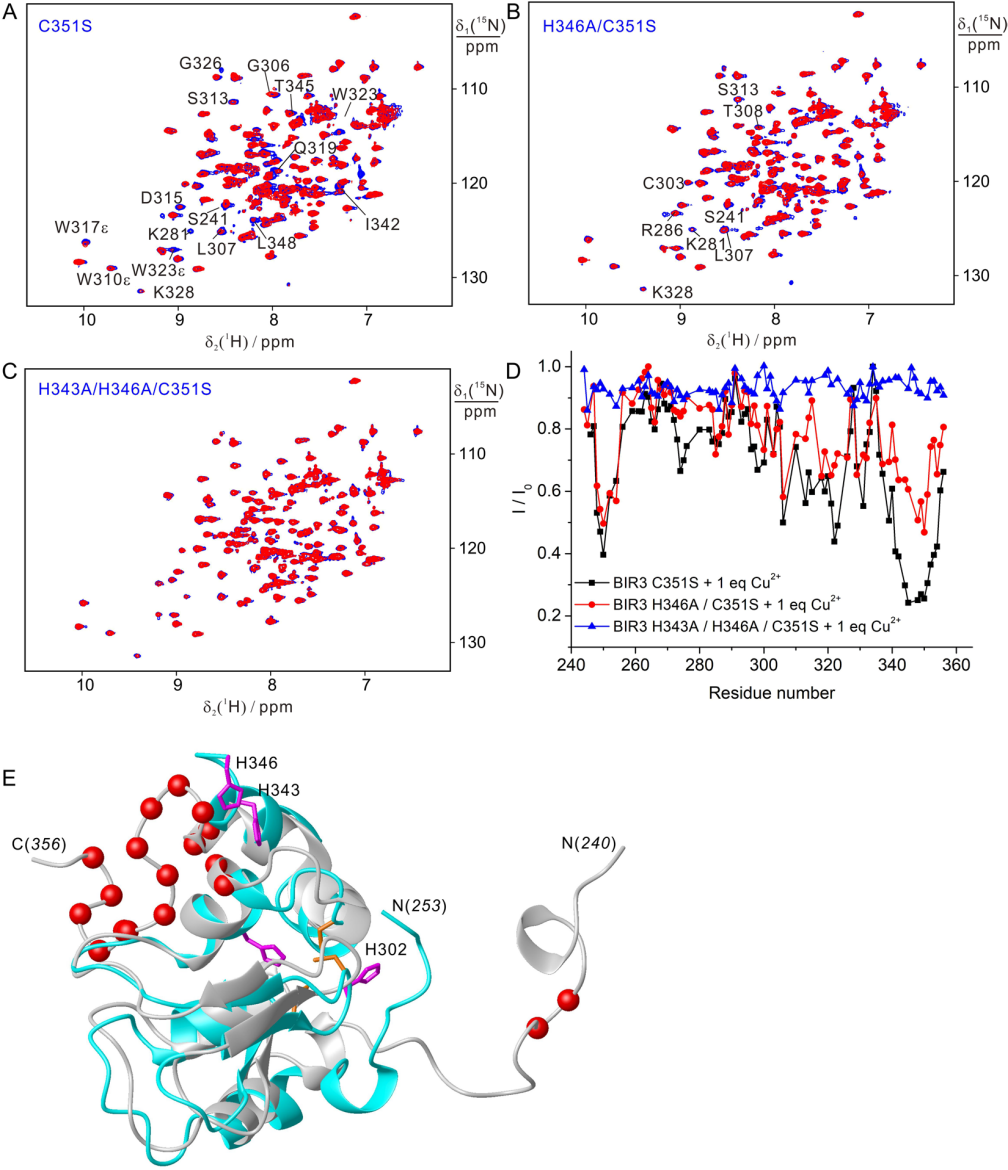
Interaction of BIR3 and its mutant with Cu(II) evaluated by ^15^N-HSQC spectra. (**A**–**C**) Superimposition of ^15^N-HSQC spectra recorded for 0.1 mM BIR3 protein before (blue) and after addition of 0.1 mM CuSO_4_ (red). (**A**) BIR3 C351S; (**B**) H346A/C351S; (**C**) H343A/H346A/C351S. (**D**) Plot of cross-peak attenuation in the ^15^N-HSQC of BIR3 mutant after addition of Cu(II) as shown in (**A**–**C**), I/I_0_, with the function of amino acid sequence, where I and I_0_ are the cross-peak intensities recorded for BIR3 mutant after and before addition of Cu(II), respectively. (**E**) Structural comparison of solution NMR structure colored in grey (PDB code: 1G3F)^17^ and crystal structure colored in cyan (PDB code: 3HL5)^18^, of which the backbone Cα atoms were labeled with red spheres for the residues I/I_0_ < 0.5. The NMR spectra were recorded in 20 mM Bis-Tris buffer, pH 6.5, at 298 K.

In addition to oxidizing C351 in formation of a disulfide bound linked dimeric BIR3, additional binding sites in BIR3 was further explored by high resolution NMR spectroscopy. C351S mutant was made to prevent from disulfide formation, and the interaction of BIR3 C351S was analyzed by NMR titration with copper sulfate. As shown in Fig. 5, Cu(II) produces significant line-broadening effects on many cross-peak of BIR3 C351S mutant. These results indicate that BIR3 contains binding sites for Cu(II). As show in Fig. 3, interaction of C351S mutant with Cu(II) did not produce dimeric BIR3, which is in contrast to the wild type form, but Cu(II) generated line broadening effects on many cross-peaks. As shown in Fig. 5, the C-terminal region containing residues N340 to V353 experienced largest PRE effects, suggesting this region contains a Cu(II) binding site. It is noted that two histidine residues, H343 and H346, are located in this region. It is known that the imidazole sidechain of histidine is a favorable Cu(II) binding ligand, and it is plausible that H343 and H346 form a Cu(II) binding motif. The coordination of H343 and H346 to Cu(II) was lately confirmed by the subsequent experiments performed on triple mutant H343A/H346A/C351S with Cu(II), which showed that the attenuation of cross-peak intensity was greatly relieved when Cu(II) was added (Fig. 5C,D).

As shown in Fig. 5D, the N-terminal region close to F250 also experienced significant PRE when Cu(II) was loaded. These residues are distant from the H343 and H346 in the solution structure of BIR3 (Figs 5E and S5)^17^, which presents different conformation from the X-ray (Fig. 5E)^18^. In the crystal structures, the N-terminal segment containing residues 253–258 presents a well conserved conformation and is close to the zinc finger. However, the NMR solution structures present opposite conformation for the N-terminal segment and the region containing residues 253–258 is further away from the zinc finger and is also distant from region containing H343 and H346. The striking differences between the X-ray and NMR structures for the N-terminal segment might be due to the sparse structural restraints in structural determinations by NMR. It is noted that mutant of H343A/H346A and H346A indeed produced chemical shift perturbations on the residues containing F250, suggesting these residues are spatially close (Fig. S5). In addition, triple mutant H343A/H346A/C351S showed no significant cross-peak attenuation, indicating that H343 and H346 are the major binding sites for Cu(II). Taken together, the decreased intensity for the N-terminal residues vicinal to F250 in Cu(II) binding is very likely due to the PRE effects caused by Cu(II), indicating the N-terminal segment is vicinal to the Cu(II) binding site in last helix containing H343, in line with the BIR3 structures as determined by X-ray crystallography^18^.

### Interaction of BIR3 with Cu(I)

#### Binding of C351 to Cu(I) results in oligomerization of BIR3

**Figure 6:**
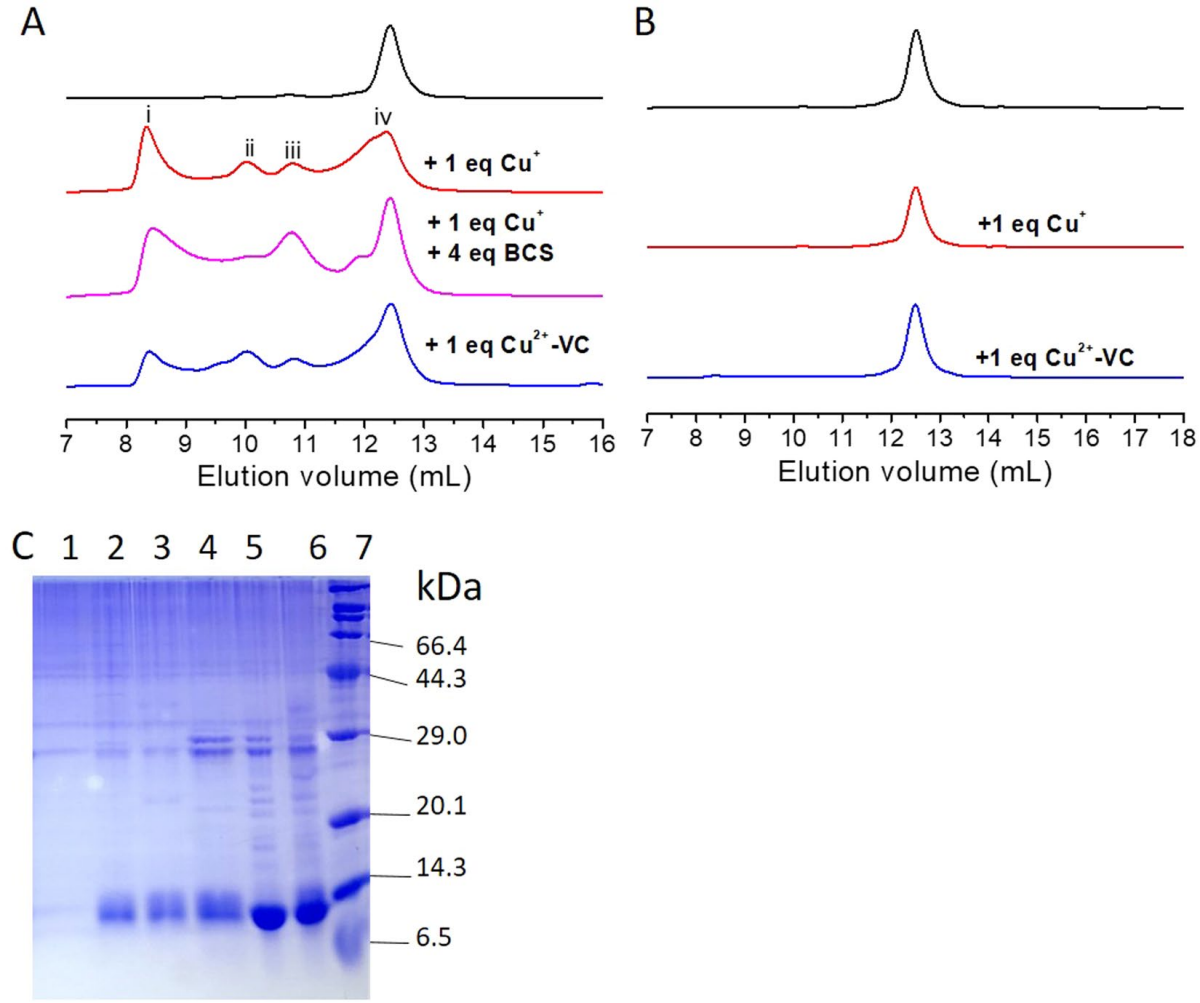
Interaction of 0.5 mM BIR3 or its C351S mutant with Cu(I). (**A**) SEC experiment performed for the reaction mixture of free BIR3 (black), BIR3 and 1 eq. Cu(I) (red), the mixture of BIR3 and 1 eq. Cu(I) treated with 4 eq. BCS (magenta), BIR3 and 1 eq. Cu(II)-VC (blue), respectively. (**B**) SEC experiment performed for the reaction mixture of free BIR3 C351S mutant (black), BIR3 C351S and 1 eq. Cu(I) (red), the mixture of BIR3 C351S and 1 eq. Cu(II)-VC (blue). (**C**) SDS-PAGE results run for the fractions from the SEC experiment of 0.5 mM BIR3 and 0.5 mM [Cu(CH_3_CN)_4_][PF_6_] from the left to right (also in Fig. S3): Lanes 1–4 lanes are the fractions of i to iv, respectively; 5: free BIR3; 6: mixture of BIR3 and 1 eq. Cu(I); 7: molecular weight marker.

To characterize the interaction of BIR3 with Cu(I), the mixture of copper sulfate with ascorbic acid (also known as vitamin C, VC) (made as molar ratio of [Cu^2+^]/[VC] = 1:9) and [Cu(CH_3_CN)_4_][PF_6_] was used, respectively. The interaction of Cu(II)-VC and [Cu(CH_3_CN)_4_][PF_6_] with BIR3 was first evaluated by size exclusion chromatography (SEC) experiment. As shown in Fig. 6, both Cu(II)-VC and [Cu(CH_3_CN)_4_][PF_6_] generated, in addition to a fraction of dimeric BIR3, multiple species that have larger molecular weights than monomeric BIR3. It is noted that the larger-molecular-weight fraction was not observed in the mixture of Cu(II) and BIR3 as shown in Fig. 3. Because Cys351 can be readily oxidized by Cu(II) in formation of a dimeric BIR3, wild type BIR3 and C351S mutant were thus used to assure whether C351 directly binds to Cu(I). Notably, the SEC data indicated that no fractions with significantly larger molecular weights than BIR3 were determined for BIR3 C351S mutant after treatment either by Cu(II)-VC or [Cu(CH_3_CN)_4_][PF_6_] (Fig. 6). Our results suggest that Cys351 binds to Cu(I) and over one BIR3 molecules are involved in Cu(I) binding to form oligomeric BIR3-Cu(I) complex (high concentration of protein results in precipitates in solution). In contrast to oxidation of C351 by Cu(II), Cu(I) binds to sulfur atom of C351. We concluded that Cys351 is the key residue in association with Cu(I).

Since BIR3 contains a zinc finger motif, it is of interest to understand whether Cu(I) is able to replace zinc ion in the zinc finger. Firstly, ^15^N-Cys labeled BIR3 was expressed and purified. Titration of [Cu(CH_3_CN)_4_][PF_6_] into the solution of BIR3 in 20 mM Bis-Tris buffer at pH 6.5 did not produce significant chemical shift perturbations or cross-peak intensity attenuations on the zinc binding motif containing C300, C303 and C327. Notably, cross-peak of C351 was decreased and a new cross-peak was observed after treatment with [Cu(CH_3_CN)_4_][PF_6_]. The new cross-peak remained unchanged after addition of 4 equivalents of bathocuproine disulfonic acid (BCS, a strong cuprous chelator), but it is diminished by addition of DTT (6 eq). These results showed that Cu(I) could not replace zinc ion from the BIR3. In addition, BIR3 C351 binds to Cu(I) with a higher affinity than BCS.

#### BIR3 has two additional main Cu(I) binding sites: H302, as well as H343 and H346

**Figure 7:**
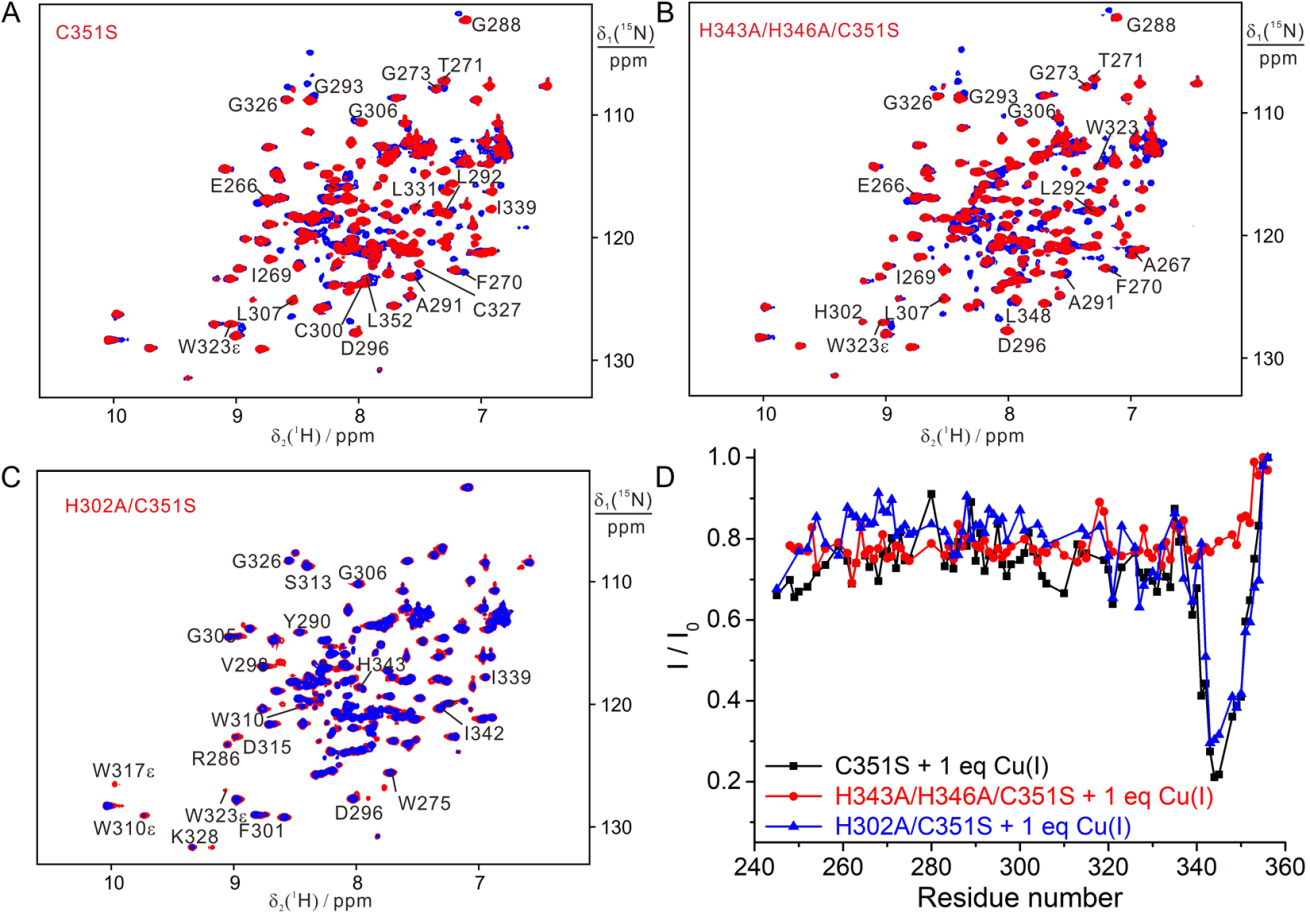
Interaction of BIR3 mutant with Cu(I) by ^15^N-HSQC experiment. Superimposition of ^15^N-HSQC spectra recorded for 0.15 mM BIR3 mutant in the absence (red) and presence of 1 eq [Cu(CH_3_CN)_4_][PF_6_](blue) in 20 mM Bis-Tris buffer, pH 6.5. (**A**) BIR3 C351S; (**B**) BIR3 H343A/H346A/C351S; (**C**) H302A/C351S. (**D**) Plot of cross-peak intensity ratio of I/I_0_ with the function of amino acid, where I and I_0_ are the cross-peak intensities in ^15^N-HSQC spectra recorded for BIR3 in the presence and absence of [Cu(CH_3_CN)_4_][PF_6_], respectively, as shown in **A** to **C**.

As we have shown that C351 is the key binding site for Cu(I), and the experiments to delineate whether additional binding sites exist in BIR3 were performed. Titration of Cu(I) into the solution of BIR3 C351S mutant generated many new cross-peaks in the ^15^N-HSQC spectrum. In addition, many cross-peaks decreased gradually in peak intensities as shown in Fig. 7. The ^15^N-HSQC spectrum of ^15^N-Cys labeled BIR3 C351S mutant indicated that addition of Cu(I) produced two new cross-peaks in addition to the C300, C303 and C327 in the zinc finger region (Fig. S6), suggesting Cu(I) might be localized in this region. In the BIR3 structure one potential Cu(I) binding ligand is the sidechain of H302 that is close to the zinc finger region. It is likely that residue C303 and C327 might participate in coordination to Cu(I) together with H302, but the exchange between Cu(I) bound and free protein is slow in the NMR spectra and the binding affinity is weaker than BCS, since the new cross-peaks disappeared after addition of 4 eq. BCS. The coordination of H302 to Cu(I) was lately confirmed by titration of H302A/C351S mutant with Cu(I), of which no new-cross peaks were produced as shown in Fig. 7C. It is noted that this interaction between H302 with Cu(I) does not break the zinc binding and no zinc ion was replaced by Cu(I) in the interaction.

Addition of Cu(I) into the solution of BIR3 also caused cross-peak intensity attenuations for the residues containing H343 and H346, which was similar to Cu(II) binding. Cu(I) is diamagnetic and it does not produce PRE effects when bound to protein, which differs greatly from Cu(II). However, no observable cross-peak attenuations were determined for these residues when Cu(I) was added to the solution of triple mutant H343A/H346A/C351S, suggesting that H343 and H346 also binds to Cu(I) in addition to Cu(II). The decreased cross-peak intensity of BIR3 C351S mutant in association with Cu(I) is probably due to conformational exchange in formation of Cu(I) complex, which increases the linewidth of cross-peaks.

### Binding affinity comparison of BIR3 with Cu(II) and Cu(I) at multiple binding sites

#### H343 and H346 have higher binding affinity for Cu(I) than Cu(II)

**Figure 8:**
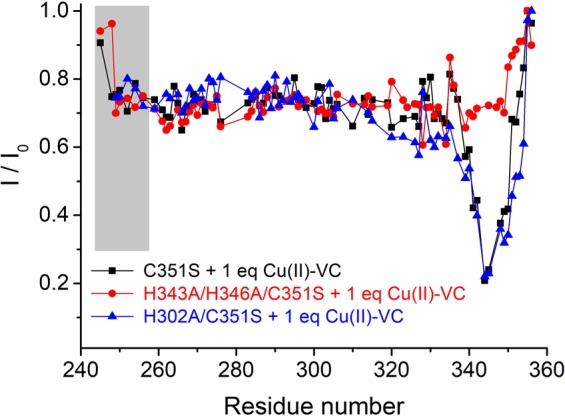
Interaction of BIR3 mutant with Cu(II) by ^15^N-HSQC experiment. Plot of cross-peak intensity ratio of I/I_0_ with the function of amino acid sequence, where I and I_0_ is cross-peak intensity ratio in the ^15^N-HSQC spectra recorded for 0.1 mM BIR3 mutant in the presence and absence of 1 eq. Cu(II)-VC, respectively.

As discussed above, H343 and H346 in BIR3 binds to not only Cu(II) and but also Cu(I), it is therefore interesting to compare which oxidation state of copper is more favorable. As shown in Fig. 5, binding to Cu(II) by H343 and H346 resulted in additional cross-peak attenuations for several residues in the N-terminal segments vicinal to F250. We showed that the N-terminal segment containing F250 is spatially close to the Cu(II) binding site in the last helix containing H343 and H346 (Fig. 5E). Therefore, one would compare the binding affinity of H343 and H346 for Cu(II) and Cu(I) by measuring the cross-peak intensities for these residues close to F250. This is because if H343 and H346 prefers to Cu(II) than Cu(I), the residues close to F250 will experience line-broadening effects due to PRE effects. If H343 and H346 prefers to Cu(I) other than Cu(II), there will be no much line-broadening effects. The mixture of Cu(II) and VC is an ideal indicator to monitor the binding strength of BIR3 for Cu(II) or Cu(I). As shown in Fig. 8, addition of the mixture of Cu(II)-VC into the solution of BIR3 C351S produced negligible PRE effects on the N-terminal residues containing F250 (structure shown in Fig. 5E), indicating that Cu(II) was reduced to Cu(I) in the interaction with this protein in the presence of VC. These results are similar to the interaction with [Cu(CH_3_CN)_4_][PF_6_] (Fig. 7D). Taken together, we conclude that H343 and H346 form more stable complex with Cu(I) than Cu(II) in the presence of VC.

#### Comparison of Cu(I) binding affinities at different binding sites in BIR3

It is showed that BIR3 have three major binding sites for Cu(I) from the above experimental data. These three binding motifs are composed by H302 (site 1), H343 and H346 (site 2), and C351 (site 3), respectively. The binding affinities of Cu(I) by H302, H343 and H346 were first evaluated by BCS. 0.1 mM BIR3 C351S was first mixed with 0.1 mM Cu-VC, and the reaction mixture was then incubated with 0.4 mM BCS for 10 h at room temperature. Accordingly, a number of ^15^N-HSQC spectra were recorded to monitor the reaction. As shown in Fig. S7, the new cross-peaks generated by addition of Cu(I) into the solution of BIR3 C351S mostly disappeared after treatment with four equivalents of BCS. In addition, the missing cross-peaks containing H343 and H346 reappeared after treatment with BCS (Fig. S7). In comparison of the cross-peak intensity ratio of I/I_0_, of which I_0_ and I are the cross-peak intensities recorded for free protein and its mixture with Cu-VC and BCS, it showed that residues containing H343 and H346 are nearly recovered after treatment with BCS (Fig. S7), indicating that no Cu(I) was bound to the protein in the presence of BCS. Taken together, we conclude that the Cu(I) binding site either H302 or H343 and H346 has weaker binding affinity for Cu(I) than BCS.

Gradual addition of Cu(I) into the solution of BIR3 C351S only produced cross-peak intensity attenuations for the residues containing N340-E350, whereas no new-cross peaks were produced during titration of Cu(I) up to 1 eq (Fig. S8). These results suggest H343 and H346 have higher affinity for Cu(I) than H302. As we have found out that C351 forms stable Cu(I) complex (Fig. 6), excess of BCS (up to 4 eq) could not regenerate free BIR3. Taken together, one can conclude that the multiple binding sites in BIR3 for Cu(I) follows the binding affinity order as C351 > H343 and H346 > H302.

## Conclusions

**Figure 9:**
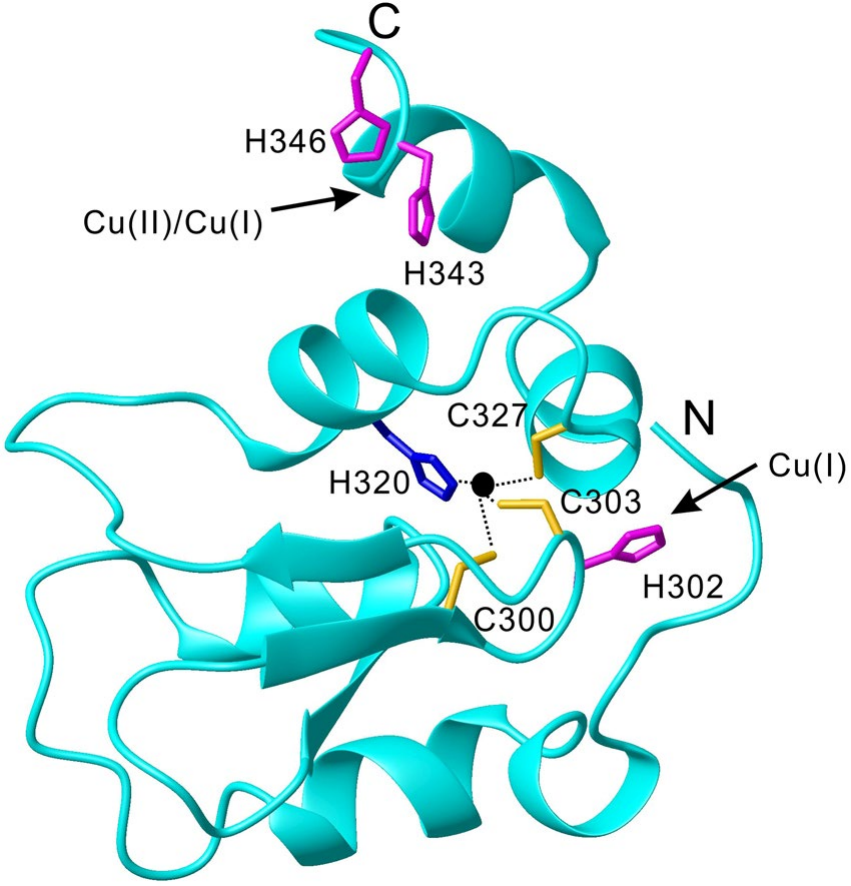
Structural representative of multiple binding sites in BIR3 for copper ion (PDB code: 3HL5)^18^. Zinc ion is labeled as black sphere in the zinc finger motif, and the flexible C351 that can be oxidized by Cu(II) and also binds to Cu(I) is absent in the crystal structure.

In the present study we extensively characterized the interaction of BIR3 and its mutant with copper ion using several biophysical methods including high resolution NMR, SEC and mass spectrometry. These results showed that BIR3 presents a multifaceted approach in association with copper ion (Fig. 9). C351 in the C-terminal domain of BIR3 has low redox potential and it is readily oxidized by Cu(II) in formation of dimeric BIR3 in solution. In cell lysates, treatment of BIR3 with Cu(II) results in formation of disulfide bond bridged BIR3-GSH complex. BIR3 is able to form stable complex with Cu(II) and Cu(I). BIR3 has one major Cu(II) binding site composed by H343 and H346. Notably, H343 and H346 also binds to Cu(I) in formation of more stable complex than Cu(II) as evidenced by PRE evaluation. Moreover, C351 binds Cu(I) tightly and form most stable Cu(I) complex than H302, H343 and H346. C351 binds Cu(I) more tightly than BCS, where the Cu(I) complexes with H302, H343 and H346 are less stable than with BCS. The binding affinity of Cu(I) in BIR3 decreases following the order of C351, H343 and H346, and H302. In total, BIR3 has three major Cu(I) binding sites which is surprising at first glance. More interestingly, these interactions of BIR3 with either Cu(II) or Cu(I) are unable to replace Zn(II) in the zinc finger motif as evidenced in the ^15^N-HSQC spectra as well as atomic absorption analysis (Table S1).

The low redox potential of C351 is similar to the recent report of C12 in BIR1^14^, suggesting the potential physiological roles in resistance of oxidation stress in tumor cells where XIAP is highly expressed. It is interesting to know that H343 and H346 in the last helix in BIR3 are able to bind both Cu(II) and Cu(I) but with varied binding affinities. This multifaceted binding whether correlates with the copper homeostasis in cells mediated by COMMD1 is highly interesting to understand, since Cu(II) binding of COMMD1 were proposed^15^. Our findings of BIR3 interacting both Cu(II) and Cu(I) in several major binding sites and with diverse association affinities sheds light on the understanding of copper interactions involved in BIR domains of XIAP. However, whether synergetic corporations of three BIR domains as well as UBA and RING domains in the process of copper homeostasis in cells involved in XIAP remain unclear and further research on this direction is highly desirable.

## Experimental Section

### Protein expression and purification

Following the previous report^17^, the cDNA encoding the sequence, containing residues 241–356 in XIAP was amplified and cloned into the pET-3a expression vector. The C351S, H302A/C351S, H343A/H346A/C351S and H346A/C351S mutants were made by PCR-based quick-change mutagenesis method and all plasmids were analysed by DNA sequencing. The expression vector was transformed into *Escherichia coli* BL21(DE3) Codon Plus strain and single colonies were selected for ampicillin and chloramphenicol resistance.

^15^N-labeled or ^13^C and ^15^N-labeled proteins were overexpressed in M9 medium following an optimized high cell-density protocol^19^. The bacteria were first grown in LB medium. At cell density at OD 600 nm about 0.6, the cells were gently collected and washed with MilliQ water. The cells were then transferred in the M9 medium containing 160 mM Na_2_HPO_4_, 40 mM KH_2_PO_4_, 2 mM MgSO_4_, 0.1 mM CaCl_2_, a trace metal mixture^20^, 1% ^12^C-glucose (or ^13^C-glucose for ^13^C labeling), ^15^NH_4_Cl (1 g/mL) and 0.1 mM ZnCl_2_. The cells were allowed to recover by incubation at 37 °C for 1 h, and the protein expression was then induced by addition of 1 mM isopropyl β-D-1-thio-galactopyranoside (IPTG). After 10 h incubation the cells were collected and resuspended with 20 mM PBS buffer at pH 8.0. The SDS page gel indicated that BIR3 was precipitated in the inclusion bodies and solubilized also in the supernatant during the over-expression. The supernatant and cell lysates were combined, and mixed with 8 M urea, 3 mM dithiothreitol (DTT) and 20 mM PBS (pH 8). The denature and refold process was performed following the previous report^14^. Pure protein was achieved through DEAE column followed by Superdex-75 gel filtration, and the buffer was then exchanged with 20 mM Bis-Tris at pH 6.5.

^15^N-Cys labeled BIR3 was expressed using the published protocol^21^ with following modifications. The protein was expressed in the optimized high density M9 media with 0.8 g/L ^15^N-Cys, 1 g/L other amino acid, 2 g/L glucose and 0.1 g/L ^14^NH_4_Cl. Purification was performed as above.

### NMR experiments and protein assignment

All NMR spectra were recorded at 298 K on a Bruker AV600 NMR spectrometer equipped with a QCI-cryoprobe unless noted elsewhere. The triple-resonance NMR experiments were performed on ^13^C, ^15^N,-BIR3 C351S mutant with a concentration of 0.5 mM in 20 mM Bis-Tris buffer at pH 6.5. All the NMR spectra were processed with the Bruker Topspin 2.1 and analyzed using NEASY program in CARA package^22^ and Sparky^23^.

The backbone of BIR3 was assigned on analysis triple resonances of 3D HNCA, HNCACB and CBCA(CO)NH and 3D NOESY-^15^N-HSQC spectrum as well as the reported solution^24,17^ and X-ray^18^ structures of BIR3.

### In-cell *E*. *coli* NMR experiment

The protocol was similar to the previous report^14^. The expression system was the same as described above, and the cells were first grown in 600 mL LB. At OD A600 0.8, the cells were gently collected by centrifuge (3000 rpm for 10 minutes). The cells were first washed with MilliQ water and then resuspended in 120 mL M9 medium with ^15^N-NH_4_Cl as sole nitrogen source. After 1 h the cells were induced with 1 mM IPTG and 0.1 mM ZnCl_2_ was added during the protein overexpression. After about 5 h, a 20 mL aliquot was pelleted at 3000 rpm for 10 min at 4 °C. The cell pellets were resuspended in 0.5 mL 20 mM Bis-Tris, pH 6.5 and 10% D_2_O for NMR measurement.

After in-cell NMR measurement, the cells were removed by centrifuge at 5000 rpm for 5 min at 4 degree and the supernatant was checked for protein leakage by NMR.

### Preparation of bacterial cell lysates

*E*. *coli* cells were grown in 600 mL LB media and then in 100 mL M9 medium containing ^15^NHCl_4_ as sole nitrogen source as described above. The cells were induced by 1 mM IPTG and left grown for about 6 h. Then the cells were collected and washed with lysate buffer (20 mM Bis-Tris, pH 6.5 in the absence of DTT or TCEP). The cells were mixed with 2 mL lysate buffer and the mixture was sonicated in an ice bath. The solution was centrifuged at 12000 rpm for 15 min at 4 °C and the supernatant was carefully transferred to an NMR tube for measurement.

The cell lysates used for crowding media were prepared by growing cells in LB media.

### Interaction of BIR3 and its mutant with copper ion

The interactions of BIR3 and BIR3 mutants with copper (II) were characterized using high resolution NMR together with other biophysical techniques. ^15^N-HSQC spectra were recorded with addition of copper sulfate (in 10 mM stock solution) into 0.15 mM BIR3 or the mutant. A number of ^15^N-HSQC spectra at the molar ratio of [Cu^2+^]/[protein] 0, 0.2, 0.6, 0.8, 1.0, 1.2 and 1.6, respectively, were recorded in 20 mM Bis-Tris, pH 6.5 and at 298 K. For comparison, the mixture of ascorbic acid and copper sulfate (molar ratio 9:1) was added stepwise into the same concentration of BIR3 or its mutant, and ^15^N-HSQC spectra were recorded accordingly.

The interactions of BIR3 and its mutants with copper (I) were analysed as below. The BIR3 protein or its mutant was titrated with tetrakis (acetonitrile) copper (I) hexafluorophophate, [Cu(I)(CH_3_CN)_4_][PF_6_] (50 mM stock solution in acetonitrile). The samples of 0.15 mM ^15^N-labeled BIR3 proteins in the absence and presence of 0.2, 0.5, 0.8, 1.0, 1.4 and 2.0 equivalent of Cu(I) were made in a glove box without oxygen, respectively, and the respective ^15^N-HSQC spectrum was recorded.

The mixture of copper sulfate and ascorbic acid (molar ratio [Cu(II)]/[VC], 1:9, and the pH was adjusted to 6.5), Cu(II)-VC, was prepared and used to differentiate the binding affinity of BIR3 with Cu(II) and Cu(I).

To evaluate the binding affinity of BIR3 with Cu(I), bathocuproine disulfonic acid (BCS) (made in 20 mM stock and pH was adjusted to 7) or GSH was titrated to the mixture of BIR3 with Cu(I), respectively, and the ^15^N-HSQC spectrum was recorded accordingly.

### Size-exclusion chromatography (SEC) experiment

The 0.12 mL BIR3 protein (wild type and C351S) at concentration of 0.5 mM in 20 mM Bis-Tris at pH 6.5 was mixed with one equivalent of copper sulfate, mixture of Cu(II)-VC, or [Cu(CH_3_CN)_4_][PF_6_], respectively. The reaction mixture was incubated at room temperature for 30 minutes and then centrifuged. The supernatant was monitored by Gel-filtration column and the fractions were further characterized by MALDI-TOF spectrometry and SDS page gel.

## Supplementary information

supporting information
