# Supplementary material for: Third BIR domain of XIAP binds to both Cu(II) and Cu(I) in multiple sites and with diverse affinities characterized at atomic resolution
Source: Sci Rep. 2019 May 15;9:7428. doi: 10.1038/s41598-019-42875-7 (PMC6520397; doi:10.1038/s41598-019-42875-7)
Supplement: Supplementary file 1 — supporting information [file 41598_2019_42875_MOESM1_ESM.pdf]

Third BIR domain of XIAP binds to both Cu(II) and Cu(I) in multiple sites and with diverse affinities characterized at atomic resolution

Shen-Na Chen, Tian Fang, Jing-Yang Kong, Bin-Bin Pan, and Xun-Cheng Su

State Key Laboratory and Research Institute of Elemento-organic Chemistry, College of Chemistry, Collaborative Innovation Center of Chemical Science and Engineering (Tianjin), Nankai University, Tianjin 300071, China.

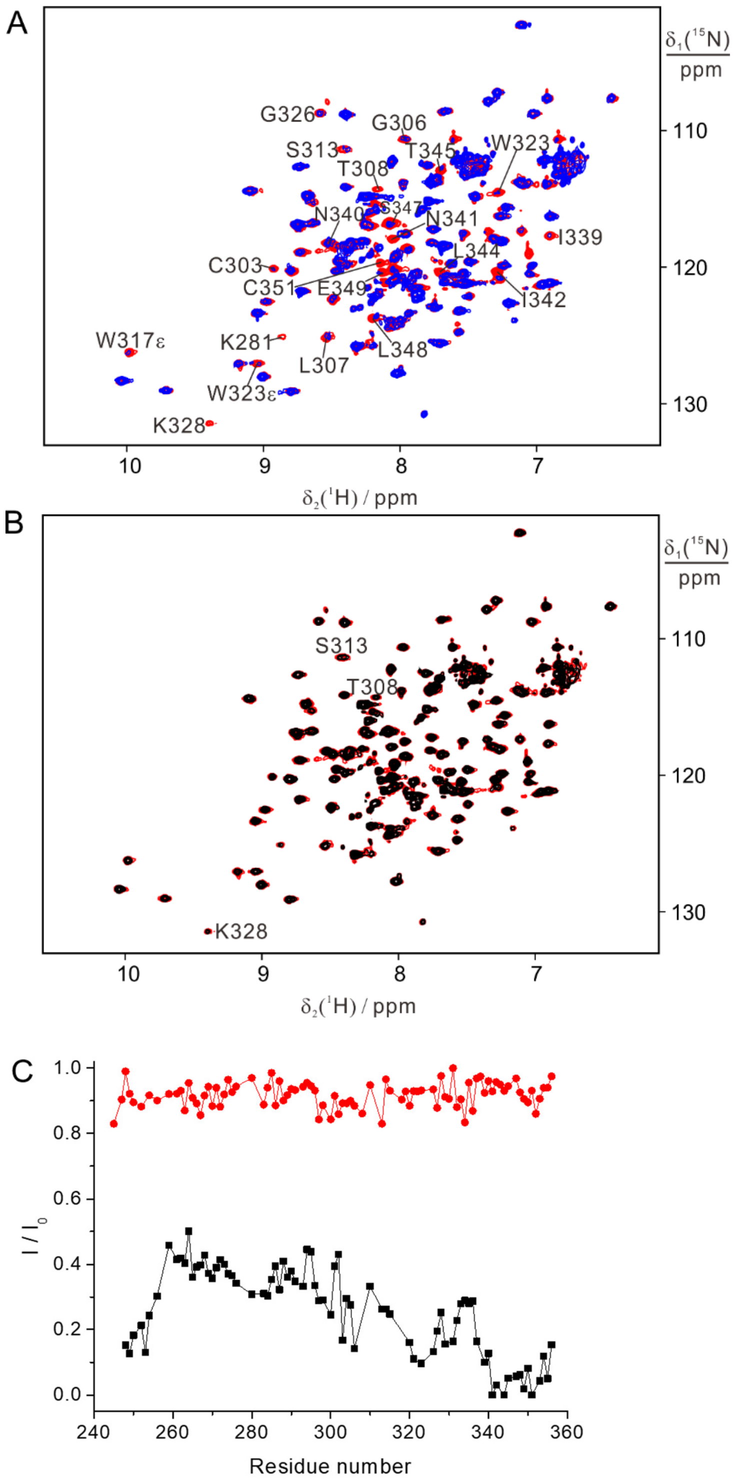

Figure S1. Interaction of BIR3 with $CuSO_4$ characterized by ^{15}N-HSQC spectra. A) Superimposition of 0.1 mM BIR3 before (red) and after (blue) addition of 0.1 mM $CuSO_4$ in 20 mM Bis-Tris buffer, pH 6.5, at 298K. B) Superimposition of 0.1 mM BIR3 before (red) and after (black) addition of 0.1 mM $CuSO_4$ and then 0.6 mM DTT in 20 mM Bis-Tris buffer, pH 6.5, at 298K. C) Plot of cross-peak intensity ratio, I/I_0, with the function of amino acid sequence. I_0 is the cross-peak intensity in the ^{15}N-HSQC spectra recorded for 0.1 mM BIR3, and I is the cross-peak intensity in the ^{15}N-HSQC spectra recorded for the mixture of 0.1 mM BIR3 and 0.1 mM $CuSO_4$ (black) or the mixture of 0.1 mM BIR3, 0.1 mM $CuSO_4$ and 0.6 mM DTT.

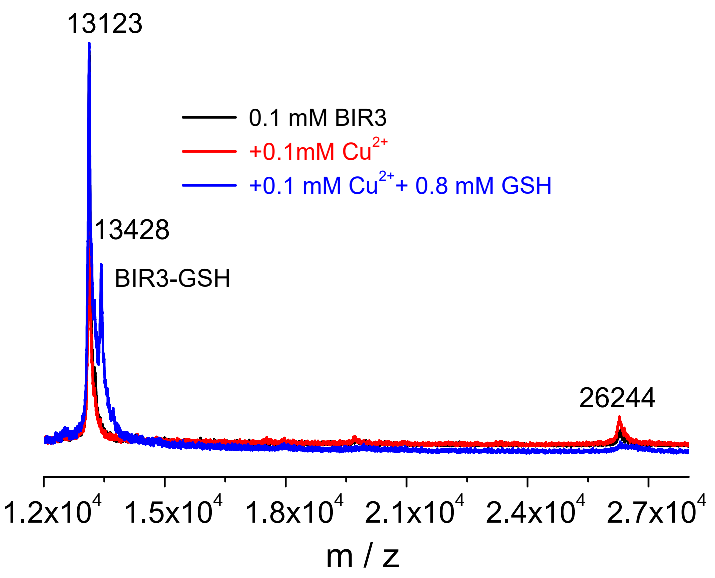

Figure S2. MALDI-TOF mass spectrometry recorded for BIR3 in the absence and presence of Cu(II): free 0.1 mM BIR3 (black), 0.1 mM BIR3 and 0.1 mM $CuSO_4$ (red); 0.1 mM BIR3 and 0.1 mM $CuSO_4$ after treatment with 0.8 mM GSH (blue). The molecular weight of free BIR3 is 13123 with removal of Zn(II) in the mass spectrum. The molecular weight of disulfide bond bridged BIR3 and GSH, 13428 was observed.

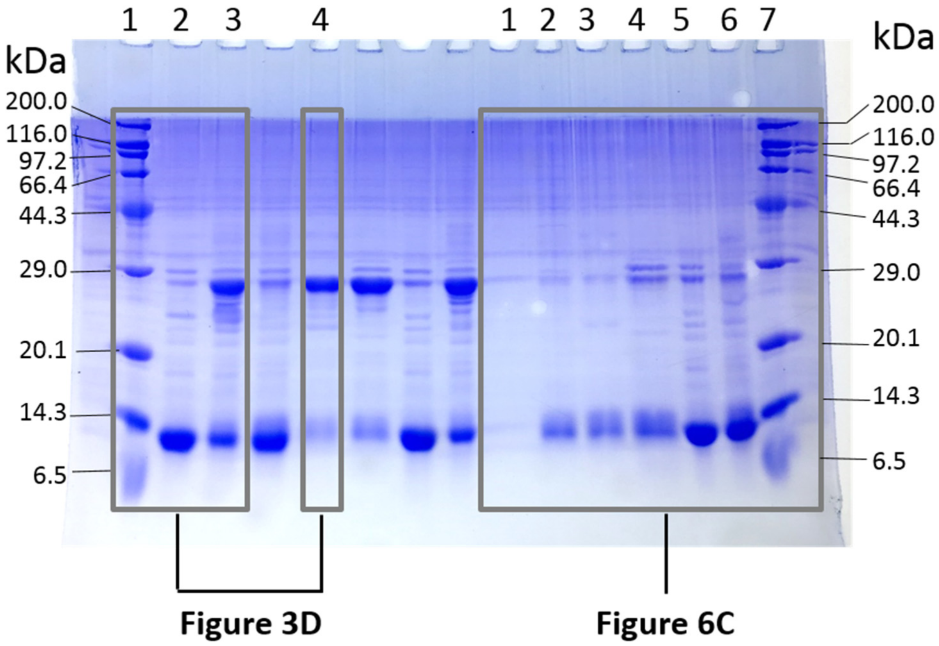

Figure S3. SDS-PAGE gel run for the different protein samples as shown in Figure 3D and Figure 6C.

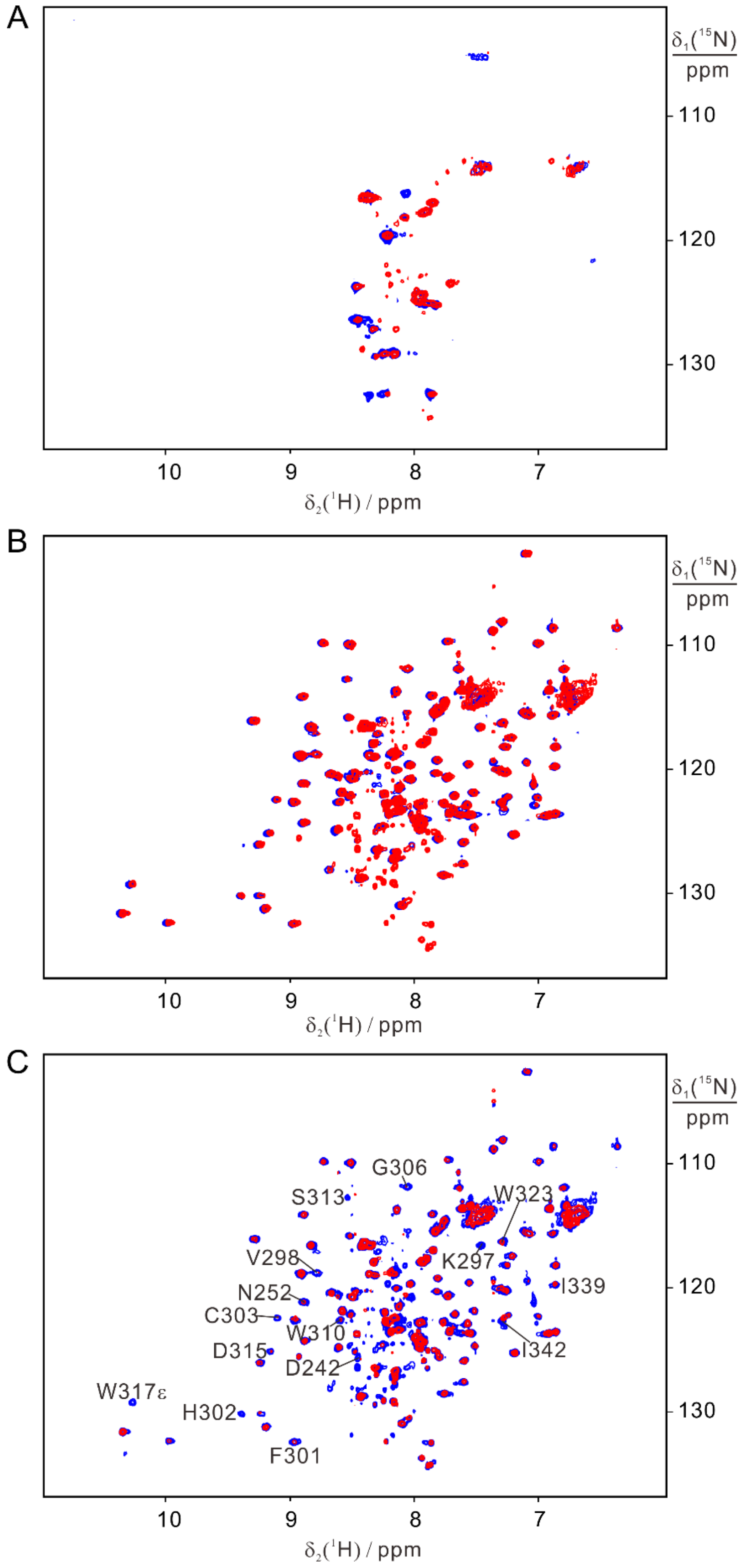

Figure S4 Comparison of BIR3 in vitro, in cell and in cell lysates using NMR methods. A) Superimposition of ^{15}N-HSQC spectra recorded for the live *E. coli* cells collected before (blue) and after (red) induction of IPTG. B) Superimposition of ^{15}N-HSQC spectra recorded for 0.1 mM BIR3 in 20 mM Bis-Tris buffer, pH 6.5 (blue) and in *E. coli* lysates (red). C) Superimposition of ^{15}N-HSQC spectra recorded for BIR3 in E coli lysates before (blue) and after (red) addition of 0.3 mM $CuSO_4$.

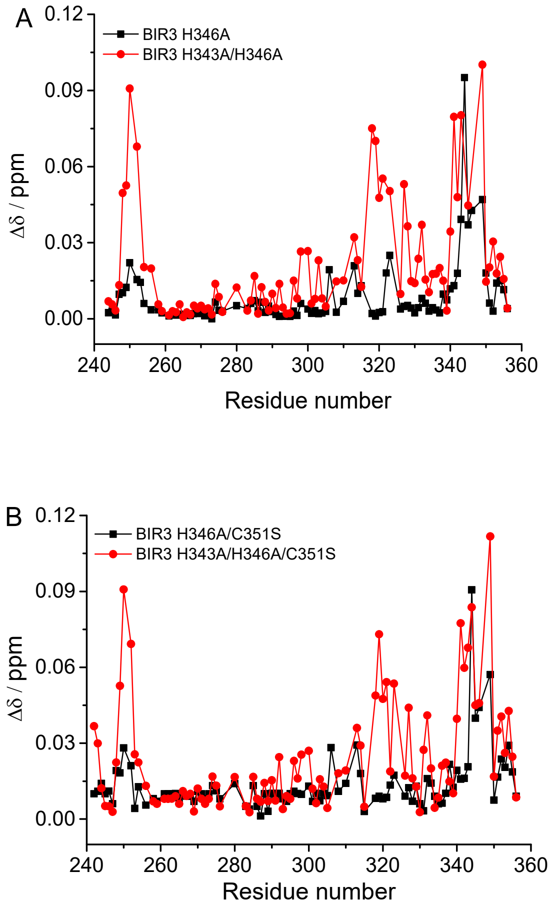

Figure S5. Chemical shift perturbations of point mutant on BIR3. (A) Plot of chemical shift perturbations caused by H343A (black) and H343A/H346A (red) mutation on BIR3 with the function of amino acid sequence. B) Plot of chemical shift perturbations caused by H346A/C351S (black) and H343A/H346A/C351S (red) mutation on BIR3 with the function of amino acid sequence. The chemical-shift differences between free protein BIR3 and its mutant were calculated as $\delta = ((\Delta\delta_H)^2 + (\Delta\delta_N/10)^2)^{1/2}$.

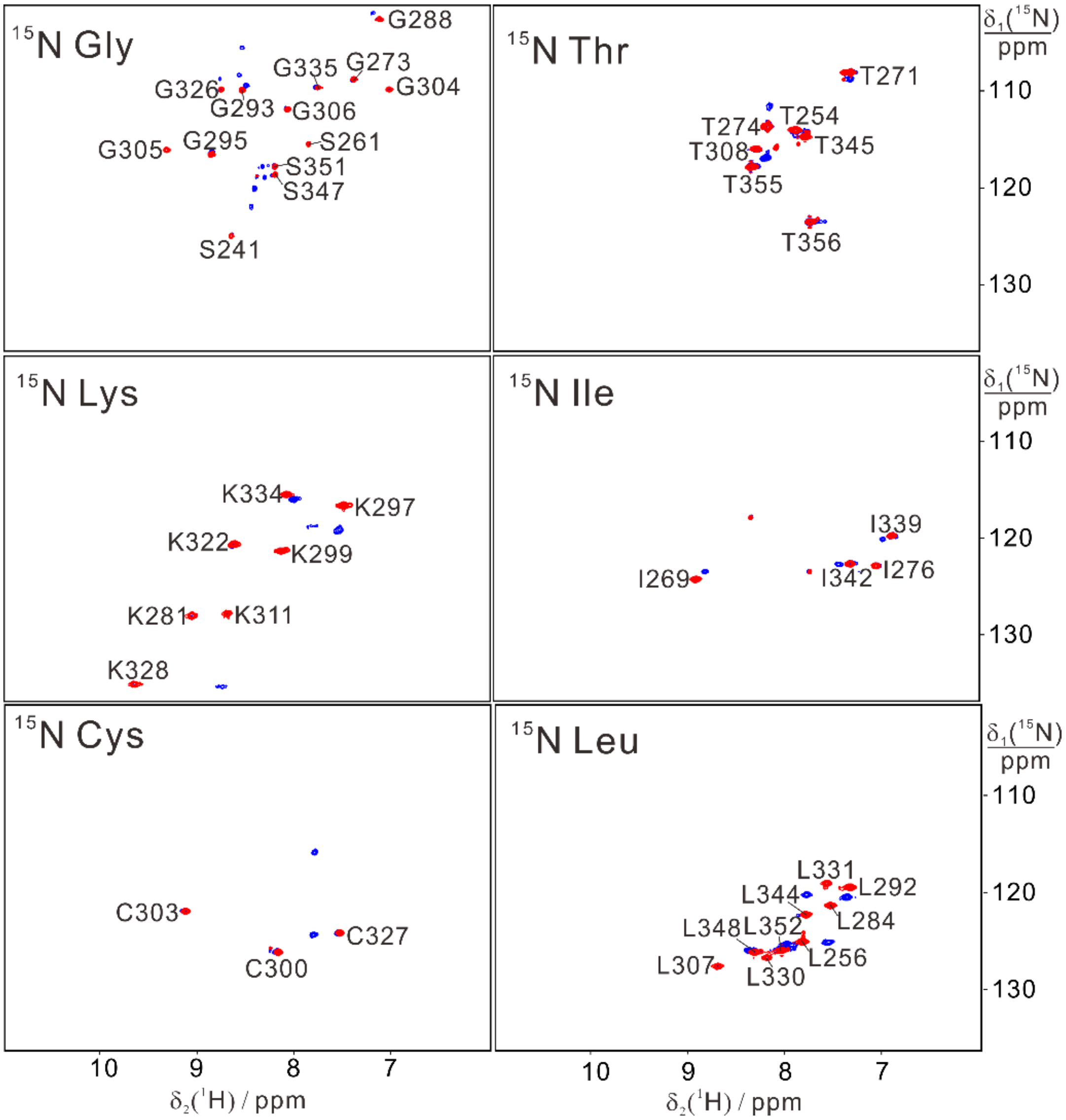

Figure S6. Interaction of BIR3 C351S mutant with Cu(I) characterized by ^{15}N-HSQC using selectively ^{15}N-labeled amino acid of BIR3. Superimposition of 0.1 mM BIR3 C351S mutant in the absence (red) and presence (blue) of 0.1 mM [Cu(CH_3CN)$_4$][PF_6]. The NMR spectra were recorded in 20 mM Bis-Tris, pH 6.5 and at 298K.

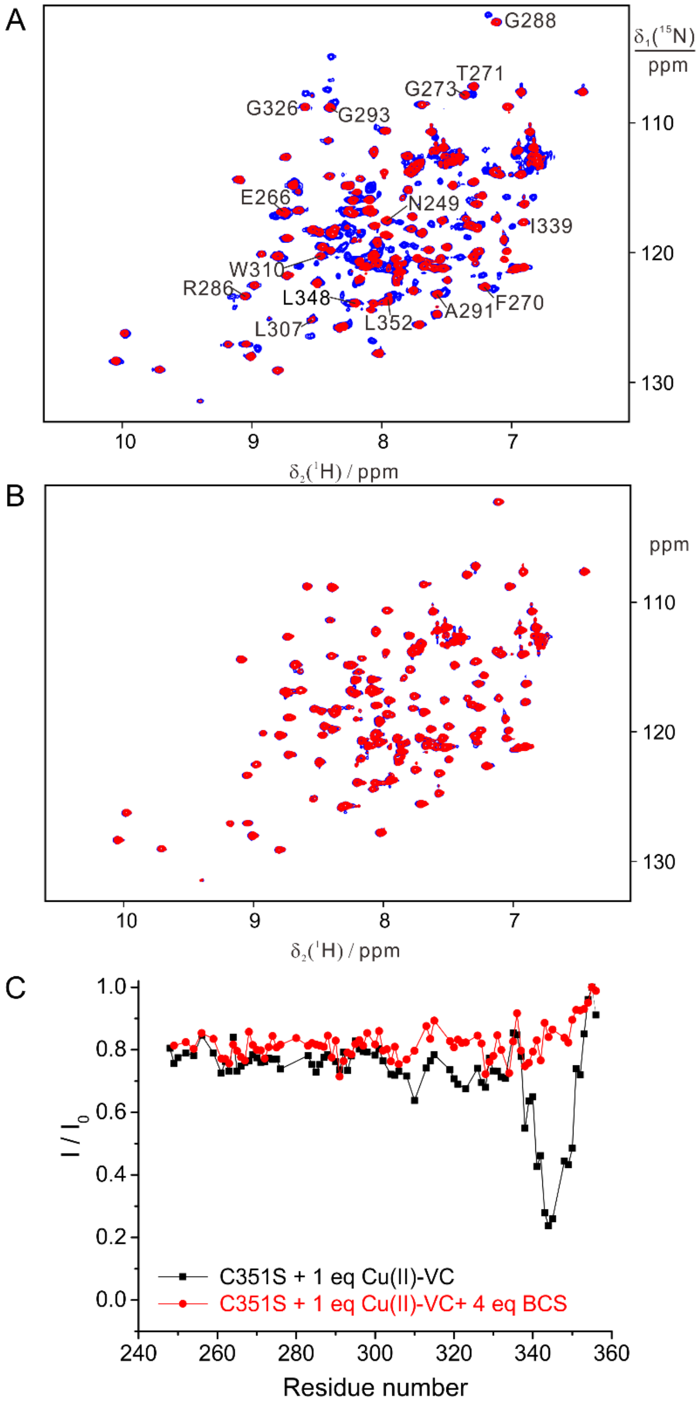

Figure S7. Binding assay of BIR3 with Cu(II)-VC characterized by ^{15}N-HSQC experiment. A) Superimposition of ^{15}N-HSQC spectra recorded for 0.1 mM BIR3 C351S in the absence (red) and presence (blue) of 0.1 mM $CuSO_4$-VC (molar ratio, [Cu]/[VC], =1:9). B) Superimposition of ^{15}N-HSQC spectra recorded for 0.1 mM BIR3 C351S (red) and the mixture of 0.1 mM BIR3 C351S, 0.1 mM $CuSO_4$-VC(molar ratio 1:9) after incubation with 0.4 mM BCS for 10 hours (blue). The NMR spectra were recorded in 20 mM Bis-Tris, pH 6.5, and at 298K. C) Plot of cross-peak intensity ratio of I/I_0 with the function of amino acids. I_0 is the cross-peak intensity in the ^{15}N-HSQC spectra recorded for 0.1 mM BIR3 C351S mutant, and I the cross-peak intensity in the ^{15}N-HSQC spectra recorded for 0.1 mM BIR3 C351S mutant in the presence of 0.1 mM Cu(II)-VC, and 0.1 mM Cu(II)-VC and 0.4 mM BCS, respectively.

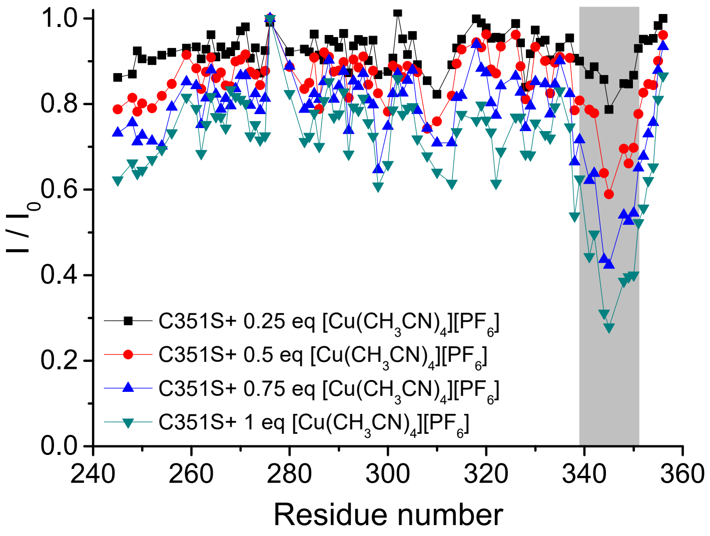

Figure S8. Plot of cross-peak intensity ratio of I/I_0 with the function of amino acids. I_0 is the cross-peak intensity in the ^{15}N-HSQC spectra recorded for 0.1 mM BIR3 C351S mutant, and I the cross-peak intensity in the ^{15}N-HSQC spectra recorded for 0.1 mM BIR3 C351S mutant in the presence of different concentrations of Cu(I) in solution.

Table S1. Atomic absorption results determined for the BIR3 treated with copper sulfate and mixture copper sulfate and VC (molar ratio 1:9).*

Sample	Cu (mM)	Zn (mM)	Protein (mM)
WT BIR3 + Cu	0.04	0.14	0.16
WT BIR3 + Cu-VC	0.05	0.16	0.16
BIR3 C351S + Cu	0.14	0.16	0.18
BIR3 C351S + Cu-VC	0.07	0.09	0.10

*The samples were prepared by mixing 0.2 mM protein and 0.2 mM copper salt, and the reaction mixture was incubated at room temperature for 5 h. Then the reaction mixture was centrifuged and the supernatant was washed twice with 20 mM Bis-Tris, pH 6.5. The left supernatant was measured at UV 280 nm for the concentration of protein and the concentrations of metal ions were determined by atomic absorption measurement.
